# Self-Management of Diabetes in Black Men: The Flint MANUP Intervention Study

**DOI:** 10.1093/geroni/igab046.1546

**Published:** 2021-12-17

**Authors:** Dana Carthron, Wayne McCullough, Samir Chatterjee, Kent Key, Kelsey Lemke, David Gordon, Gretchen Piatt, Harold Neighbors

**Affiliations:** 1 North Carolina Central University, North Carolina Central University, North Carolina, United States; 2 Michigan State University, Flint, Michigan, United States; 3 Claremont Graduate University, Claremont, California, United States; 4 University of Michigan-Flint, Flint, Michigan, United States; 5 University of Michigan-Flint, Ann Arbor, Michigan, United States; 6 University of Michigan, University of Michigan, Michigan, United States

## Abstract

The story of John Henry, the “steel-drivin’ man”, is well known to Black men in the United States. John Henry is considered a hero because he demonstrated tremendous strength and self-determination. The MANUP diabetes program used the John Henryism, defined as high-effort active coping in the face of adversity, as the basis of a diabetes intervention for Black men. MANUP conducted four community-based focus groups identifying topics of concern to Black men with type 2 diabetes (T2D). Interestingly, the men reported that high-effort active coping was crucial for successful diabetes self-management. MANUP then developed and implemented a longitudinal culturally targeted self-management program for 33 Black men with T2D in Flint, Michigan. MANUP included discussion groups, physical activity, and an app incorporating text-messaging, group-chat, and a blood glucose monitoring dashboard to improve glycemic control (A1c). This single-group, repeated measures intervention assessed A1c three times over a six-month period. Improvements in A1c were observed at: baseline – time 2: 8.9% vs 8.6%, p=0.14; time 2 – time 3: 8.6% vs 8.1%, p=0.21; and baseline – time 3: 8.9% vs 8.1%, p=0.005. After controlling for age and insulin use, the significant reduction in A1c over 6 months remained (p=0.01). These findings demonstrate that combining mobile health technology and moderate physical activity with culturally targeted discussion topics can improve T2D self-management and reduce A1c in Black men. More community-driven longitudinal intervention studies that improve diabetes self-management among Black men are needed to achieve gender and racial health equity..

